# On epidemic modeling in real time: An application to the 2009 Novel A (H1N1) influenza outbreak in Canada

**DOI:** 10.1186/1756-0500-3-283

**Published:** 2010-11-05

**Authors:** Ying-Hen Hsieh, David N Fisman, Jianhong Wu

**Affiliations:** 1 Department of Public Health and Center for Infectious Disease Education and Research, China Medical University, Taichung, Taiwan; 2 Ontario Agency for Health Protection and Promotion and Dalla Lana School of Public Health, University of Toronto, Toronto, M5T 3M7, Canada; 3 Centre for Disease Modeling, York University, Toronto, M3J 1P3, Canada

## Abstract

**Background:**

Management of emerging infectious diseases such as the 2009 influenza pandemic A (H1N1) poses great challenges for real-time mathematical modeling of disease transmission due to limited information on disease natural history and epidemiology, stochastic variation in the course of epidemics, and changing case definitions and surveillance practices.

**Findings:**

The Richards model and its variants are used to fit the cumulative epidemic curve for laboratory-confirmed pandemic H1N1 (pH1N1) infections in Canada, made available by the Public Health Agency of Canada (PHAC). The model is used to obtain estimates for turning points in the initial outbreak, the basic reproductive number (R_0_), and for expected final outbreak size in the absence of interventions. Confirmed case data were used to construct a best-fit 2-phase model with three turning points. R_0 _was estimated to be 1.30 (95% CI 1.12-1.47) for the first phase (April 1 to May 4) and 1.35 (95% CI 1.16-1.54) for the second phase (May 4 to June 19). Hospitalization data were also used to fit a 1-phase model with R_0 _= 1.35 (1.20-1.49) and a single turning point of June 11.

**Conclusions:**

Application of the Richards model to Canadian pH1N1 data shows that detection of turning points is affected by the quality of data available at the time of data usage. Using a Richards model, robust estimates of R_0 _were obtained approximately one month after the initial outbreak in the case of 2009 A (H1N1) in Canada.

## Background

Epidemics and outbreaks caused by emerging infectious diseases continue to challenge medical and public health authorities. Outbreak and epidemic control requires swift action, but real-time identification and characterization of epidemics remains difficult [1]. Methods are needed to inform real-time decision making through rapid characterization of disease epidemiology, prediction of short-term disease trends, and evaluation of the projected impacts of different intervention measures. Real-time mathematical modeling and epidemiological analysis are important tools for such endeavors, but the limited public availability of information on outbreak epidemiology (particularly when the outbreak creates a crisis environment), and on the characteristics of any novel pathogen, present obstacles to the creation of reliable and credible models during a public health emergency. One needs to look no further than the 2003 SARS outbreak, or ongoing concerns related to highly pathogenic avian influenza (H5N1) or bioterrorism to be reminded of the need for and difficulty of real-time modeling.

The emergence of a novel pandemic strain of influenza A (H1N1) (pH1N1) in spring 2009 highlighted these difficulties. Early models of 2009 pH1N1 transmission were subject to substantial uncertainties regarding all aspects of this outbreak, resulting in uncertainty in judging the pandemic potential of the virus and the implementation of reactive public health responses in individual countries (Fraser et al. [2]). Multiple introductions of a novel virus into the community early in the outbreak could further distort disease epidemiology by creating fluctuations in incidence that are misattributed to the behavior of a single chain of transmission.

We sought to address three critical issues in real time disease modeling for newly emerged 2009 pH1N1: (i) to estimate the basic reproduction number; (ii) to identify the main turning points in the epidemic curve that distinguish different phases or waves of disease; and (iii) to predict the future course of events, including the final size of the outbreak in the absence of intervention. We make use of a simple mathematical model, namely the Richards model, to illustrate the usefulness of near real-time modeling in extracting valuable information regarding the outbreak directly from publicly available epidemic curves. We also provide caveats regarding inherent limitations to modeling with incomplete epidemiological data.

The accuracy of any modeling is highly dependent on the epidemiological characteristics of the outbreak considered, and most epidemic curves exhibit multiple turning points (peaks and valleys) during the early stage of an outbreak. While these may be due to stochastic ("random") variations in disease spread, and changes in either surveillance methods or case definitions, turning points may also represent time points where epidemics transition from exponential growth processes to processes that have declining rates of growth, and thus may identify effects of disease control programs, peaks of seasonal waves of infection, or natural slowing of growth due to infection of a critical fraction of susceptible individuals. For every epidemic, there is a suitable time point after which a given phase of an outbreak can be suitably modeled, and beyond which subsequent phases may be anticipated. Detection of such "turning points" and identification of different phases or waves of an outbreak is of critical importance in designing and evaluating different intervention strategies.

## Methods

Richards [3] proposed the following model to study the growth of biological populations, where *C(t) *is the cumulative number of cases reported at time *t *(in weeks):

$$C^{\prime }(t)=rC(t)[1-{\left(\frac{C}{K}\right)}^{a}].$$

Here the prime "'" denotes the rate of change with respect to time. The model parameter *K *is the maximum case number (or final outbreak size) over a single phase of outbreak, *r *is the per capita growth rate of the infected population, and *a *is the exponent of deviation. The solution of the Richards model can be explicitly given in terms of model parameters as $C(t)=K{\left[1+e^{-r(t-t_{m})}\right]}^{-1/a}$, and the parameter *t*_m _is related to the turning point t_i _of the epidemic (or the inflection point of the cumulative case curve) by the simple formula *t_m _*= *t_i _*+ (*lna*)/*n *where *ln *denotes the natural logarithm. Using the Richard model, we are able to directly fit empirical data from a cumulative epidemic curve to obtain estimates of epidemiological meaningful parameters, including the growth rate r.

In such a model formulation, the basic reproduction number R_0 _is given by the formula *R*_0 _= *exp*(*rT*) where T is the disease generation time defined as the average time interval from infection of an individual to infection of his or her contacts. It has been shown mathematically [4] that, given the growth rate *r*, the equation *R*_0 _= *exp*(*rT*) provides the upper bound of the basic reproduction number regardless of the distribution of the generation interval used, assuming there is little pre-existing immunity to the pathogen under consideration. Additional technical details regarding the Richards model can be found in [5-7].

Unlike the better-known deterministic compartmental models used to describe disease transmission dynamics, the Richards model considers only the cumulative infected population size. This population size is assumed to have saturation in growth as the outbreak progresses, and this saturation can be caused by immunity, by implementation of control measures or other factors such as environmental or social changes (e.g., children departing from schools for summer holiday). The basic premise of the Richards model is that the incidence curve of a single phase of a given epidemic consists of a single peak of high incidence, resulting in an S-shaped cumulative epidemic curve with a single turning point for the outbreak. The turning point or inflection point, defined as the time when the rate of case accumulation changes from increasing to decreasing (or vice versa) can be easily pinpointed as the point where the rate of change transitions from positive to negative; i.e., the moment at which the trajectory begins to decline. This time point has obvious epidemiologic importance, indicating either the beginning of a new epidemic phase or the peak of the current epidemic phase.

For epidemics with two or more phases, a variation of the S-shaped Richards model has been proposed [6]. This multi-staged Richards model distinguishes between two types of turning points: the initial S curve which signifies the first turning point that ends initial exponential growth; and a second type of turning point in the epidemic curve where the growth rate of the number of cumulative cases begins to increase again, signifying the beginning of the next epidemic phase. This variant of Richards model provides a systematic method of determining whether an outbreak is single- or multi-phase in nature, and can be used to distinguish true turning points from peaks and valleys resulting from random variability in case counts. More details on application of the multi-staged Richards model to SARS can be found in [6,7]. Readers are also referred to [8,9] for its applications to dengue.

We fit both the single- and multi-phase Richards models to Canadian cumulative 2009 pH1N1 cumulative case data, using publicly available disease onset dates obtained from the Public Health Agency of Canada (PHAC) website [10,11]. PHAC data represent a central repository for influenza case reports provided by each of Canada's provinces and territories. Onset dates represent best local estimates, and may be obtained differently in different jurisdictions. For example, the province of Ontario, which comprises approximately 1/3 of the population of Canada, and where most spring influenza activity was concentrated, replaces onset dates using a hierarchical schema, whereby missing onset dates may be replaced with dates of specimen collection (if known) or date of specimen receipt by the provincial laboratory system, if both dates of onset and specimen collection are missing.

Data were accessed at different time points during the course of the "spring wave (or herald wave)" of the epidemic in May-July of 2009, whenever a new dataset is made available online by the PHAC. By sequentially considering successive S-shaped segments of the epidemic curve, we estimate the maximum case number (K) and locate turning points, thus generating estimates for cumulative case numbers during each phase of the outbreak. The PHAC cumulative case data is then fitted to the cumulative case function *C(t) *in the Richards model with the initial time *t_0 _*= 0 being the date when the first laboratory confirmed case was reported and the initial case number *C_0 _*= *C(0) *= 1, (the case number with onset of symptoms on that day). There were some differences between sequential epidemic curves in assigned case dates. For example, data posted by PHAC on May 20 indicated an initial case date of April 13, but in the June 3 data this had been changed to April 12, perhaps due to revision of the case date as a result of additional information.

Model parameter estimates based on the explicit solution given earlier can be obtained easily and efficiently using any standard software with a least-squares approximation tool, such as SAS or Matlab.

Daily incidence data by onset date were posted by PHAC until June 26, after which date only the daily number of laboratory-confirmed hospitalized cases in Canada was posted. For the purpose of comparison, we also fit the hospitalization data to the Richards model in order to evaluate temporal changes in the number of severe (hospitalized) cases, which are assumed to be approximately proportional to the total cases number. The case and hospitalization data used in this work are provided online as Additional file 1.

## Results

We fit the model to the daily datasets, acquired in real time, throughout the period under study. The least-squared approximation of the model parameter estimation could converge for either the single-phased or the 2-phase Richards models. For the sake of brevity, only four of these model fits are presented in Table 1 to demonstrate the difference in modeling results over time. The resulting parameter estimates with 95% confidence intervals (CI) (for turning point (t_i_), growth rate (r), and maximum case number (K)), time period included in the model, and time period when the data set in question were accessed, is presented in Table 1. Note that all dates in the tables are given by month/day. We also note that the CI's for R_0 _reflect the uncertainty in T as well as in the estimates for r, and does not reflect the error due to the model itself, which is always difficult to measure.

**Table 1:** Estimation results for Richards model parameters for various time periods of Canadian daily laboratory-confirmed pandemic H1N1 virus data by onset date

Time period (date posted)	Model duration	**Turning point t**_**i **_**(95% CI)**	Growth rate r (95% CI)	**Turning point t**_**i**_	**R**_**0 **_**(95% CI)**	**R**_**0**_^**#**^**(95% CI)**
4/13-5/15 (5/20)	4/13-5/6	14.70 (0.32, 29.09)	0.14 (0.12, 0.17)	4/28	1.32 (1.17, 1.46)	1.68 (1.45, 1.91)
	5/6-5/15	1.59 (0*, 4)	0.66 (0.57, 0.74)	5/8	3.49 (1.80, 5.19)	10.57 (4.79, 16.36)
4/12-5/27 (6/3)	4/12-5/27	25.70 (20.67, 30.73)	0.13 (0.10, 0.16)	5/8	1.28 (1.14, 1.42)	1.60 (1.37, 1.82)
4/11-6/5 (6/10)	4/11-5/6	17.84 (6.72, 28.97)	0.14 (0.11, 0.17)	4/29	1.30 (1.16, 1.45)	1.64 (1.40, 1.88)
	5/6-6/5	15.14 (0*, 93.02)	0.09 (0.06, 0.11)	5/22	1.18 (1.09, 1.27)	1.37 (1.22, 1.52)
4/12-6/19 (6/26)	4/12-5/4	16.85 (0*, 49.04)	0.14 (0.09, 0.19)	4/29	1.30 (1.12, 1.47)	1.63 (1.31, 1.96)
	5/4-6/19	31.15 (28.83, 33.47)	0.13 (0.11, 0.16)	6/4	1.29 (1.15, 1.53)	1.62 (1.40, 1.84)

*max(0, lower bound)

Note that all dates in the tables are given by month/day. Dates of posting are listed in parentheses. Model duration indicates whether they fit a 1-phase or 2-phase model. Note that the maximum case number is rounded off to the nearest integer. R_0_^# ^is obtained using the generation interval of T = 3.6 (2.9, 4.3) for seasonal influenza [13].

In order to compare the 1-phase and 2-phase models, we also calculate the Akaike information criterion (AIC) [12] for the first, third, and fourth sets of data in Table 1, where there is a model fit for the 2-phase model. The results, given in Table 2, indicates that whenever there is a model fit for the 2-phase model, its AIC value is always lower than that of the 1-phase model and hence compares favorably to the 1-phase model.

**Table 2:** Comparison of Akaike information criterion (AIC) values between 1-phase and 2-phase models for time periods with 2-phase model fit in Table 1

Time period (date posted)	Model	AIC
4/13-5/15 (5/20)	1-phase	352
	2-phase	163
4/11-6/5 (6/10)	1-phase	589
	2-phase	499
4/12-6/19 (6/26)	1-phase	1286
	2-phase	771

**Figure 1:**
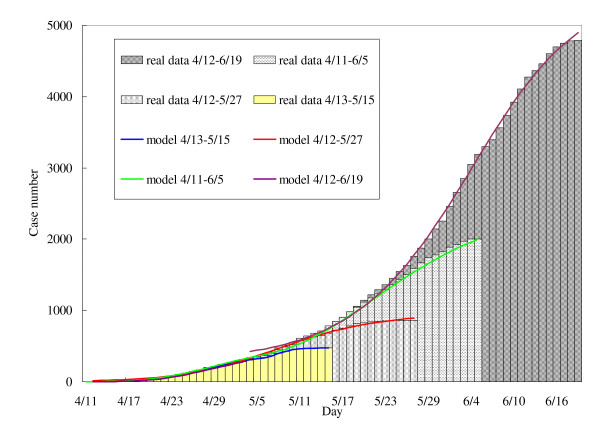
**The 2-phase Richards model for the initial phase of the influenza A (H1N1) 2009 in Canada, using daily incidence data by onset date between April 11-June 19 and posted by PHAC on June 26**.

Parameter estimates fluctuate in early datasets, and the least-squared parameter estimations diverge within and between 1-phase and 2-phase models in a manner that seems likely to reflect artifact. In particular, for the earliest model fits, using data from April 13 to May 15, the estimated reproductive number for the second phase is far larger than that obtained in the first phase, and that obtained using a single-phase model, and illustrating the pitfalls of model estimation using the limited data available early in an epidemic. Estimates stabilize as the outbreak progresses, as can be seen with the final data sets (April 11 to June 5 and April 12 to June 19). For comparison, we plot the respective theoretical epidemic curves based on the Richards model with the estimated parameters described in the table above in Figure 1.

As noted above, model can be used to estimate turning points (t_i_) and basic reproductive numbers (R_0_.), if the generation time T is know. We used T = 1.91 days (95% CI: 1.30-2.71), as obtained in [2] by fitting an age stratified mathematical model to the first recognized 2009 influenza A (H1N1) outbreak in La Gloria, Mexico. Estimates are presented in Table 1. We also conducted sensitivity analyses with R_0_^# ^calculated based on longer generation times (T = 3.6 (2.9, 4.3)) for seasonal influenza in [13] (see last column in Table 1). Excluding implausibly high estimates of R_0 _generated using initial outbreak data (April 13 to May 15), we obtain the estimates of R_0 _for the 2-phase model that range between 1.31 and 1.96.

**Figure 2:**
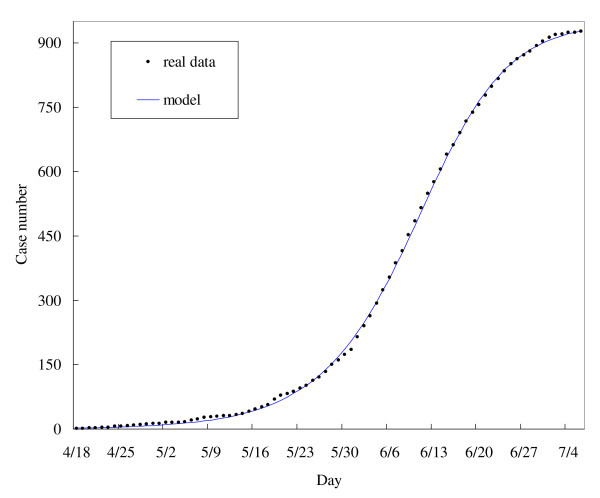
**The 2-phase Richards model for laboratory-confirmed pandemic influenza A H1N1 2009 cases hospitalized in Canada during April 18-July 6 and posted by Public Health Agency of Canada on 15 July 2009**.

Inasmuch as Richards model analyzes the general trends of an epidemic (e.g., turning point, reproductive number, etc.), it can be used to fit any epidemiological time series for a given disease process, as long as the rate of change in the recorded outcome is proportional to changes in the true number of cases. As such, for comparison, we fit our model using the time series for 2009 pH1N1 hospitalizations in Canada posted by PHAC on July 15 [11] (that last date these data were made available) (Table 3). This time series was easily fit to a one-phase model (Figure 2). Further examples of using hospitalization or mortality data to fit the Richards model can be found in [14].

**Table 3:** Comparison of estimation results of the model parameters for the 1-phase Richards model using daily laboratory-confirmed pandemic H1N1 virus hospitalization data

Time period of data (date posted)	**Estimated turning point t**_**i**_	**R**_**0 **_**(95% CI)**	**R**_**0**_^**# **^**(95% CI)**
4/18-7/6 (7/15)	June 11	1.35 (1.20, 1.49)	1.75 (1.55, 1.95)

## Discussion and Conclusions

We used the Richards model, which permits estimation of key epidemiological parameters based on cumulative case counts, to study the initial wave of 2009 influenza A (H1N1) cases in Canada. In most model fits, April 28-29 and May 4-7 were identified as early turning points for the outbreak, with a third and final turning point around June 3-5 in models based on longer time series. Although this modeling approach was not able to detect turning points using some earlier data sets (e.g., those limited to the period from April 12 to May 27), in general the turning points identified were consistent across multiple models and time series. Perhaps the most important divergence between models occurred with the detection of an April 29 turning point in the case report time series, but not in the time series based on hospitalized cases. We believe this may be attributable to the small number of hospitalizations, relative to cases, that had occurred by that date, as well as the fact that hospitalization data only became available on April 18.

The turning point can correspond to the point at which disease control activities take effect (such that the rate of change in epidemic growth begins to decline) or can represent the point at which an epidemic begins to wane naturally (for example, due to seasonal shifts or due to the epidemic having "exhausted" the supply of susceptibles such that the reproductive number of the epidemic declines below 1). This quantity has direct policy relevance; for example, in the autumn 2009 pH1N1 wave in Canada, vaccination for pH1N1 was initiated at or after the turning point of the autumn wave due to the time taken to produce vaccine; as the epidemic was in natural decline at that point, the impact of vaccination has subsequently been called into question.

**Figure 3:**
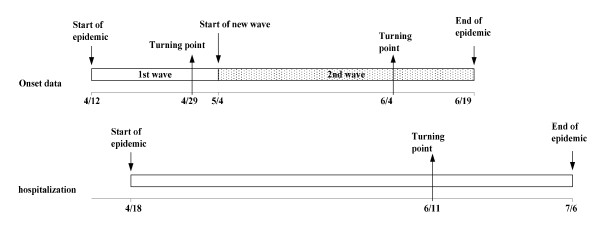
**Timelines of the 2-phase Richards model using case data of April 12-June 19 and the 1-phase model using hospitalization data**.

Although the Richards model is able to capture the temporal changes in epidemic dynamics over the course of an outbreak, it does not define their biological or epidemiological basis. As such, determining the nature of these turning points requires knowledge of "events on the ground" for correlation. We suspect that the last identified turning point (in early June) occurred in relation to intensifying requests by the Ontario Public Health Laboratory that clinicians stop routine virological testing of all individuals with respiratory illness, as well as the dismissal of schools in June. With respect to the lag between final turning points using a 2-phase model for case data (June 4) (Table 1, last line) and a 1-phase model using hospitalization data (June 11), this lag in turning points would actually be expected, due to the time from initial onset of symptoms until hospitalization, which was reported to have an interquartile range of 2-7 days in a recent study from Canada [15]. Timelines for the 2-phase model for case data of 4/12-6/19 and the 1-phase model for hospitalization data are presented graphically in Figure 3.

In addition to identifying turning points, the Richards model is useful for estimation of the basic reproductive number (R_0_) for an epidemic process, and our estimates derived using a Richards model were consistent with estimates derived using other methods. For example, our R_0 _agrees almost perfectly with that of Tuite et al., derived using a Markov chain Monte Carlo simulation parameterized with individual-level data from Ontario's public health surveillance system [16]. Our estimates of R_0 _is smaller than that derived by Fraser et al. [2] using Mexican data, but such differences could relate in part to the different age distributions of these two countries [17], and may also reflect the fact that our estimate is obtained Canadian data at a national level, while empirical Mexican estimates were based on data from the town of La Gloria with only 1575 residents.

Most epidemic curves in the early stage of a novel disease outbreak have multiple phases or waves due to simple stochastic ("random") variation, mechanisms of disease importing, initial transmission networks and individual/community behavior changes, improvements in the performance of surveillance systems, or changes in case definitions as the outbreak response evolves. However, changes in phase (signified by the presence of turning points identified using the Richards model) may also pinpoint the timing of important changes in disease dynamics, such as effective control of the epidemic via vaccination or other control measures, depletion of disease-susceptible individuals (such that the effective reproductive number for the disease decreases to < 1), or the peak of a "seasonal" wave of infection, as occurs with both seasonal and pandemic influenza. One of the advantages of the Richards model for real-time modeling is that it offers a simple means of fitting a model to cumulative case counts, which smoothes out stochastic variations in the epidemic curve.

While there are numerous published methodologies in addition to the Richards model that can be used to estimate R_0 _[4,18,19], some competing methods require more extensive and detailed data than are required to build a Richards model, which requires only cumulative case data from an epidemic curve. As we also demonstrate here, the Richards model produces fairly stable and credible estimates of reproductive numbers early in the outbreak, allowing these estimates to inform evolving disease control strategies. For example, the first estimates in Table 1, derived using early case data accessed on May 20, closely approximate our final estimates (Table 1, last row). Thus, while early estimation with the Richards model failed to correctly detect turning points or accurately estimate the final outbreak size, it was nonetheless useful for rapid estimation of R_0 _within a month of first case occurrence in Canada.

As with any mathematical modeling technique, the approach presented here is subject to limitations, which include data quality associated with real-time modeling (as data are often subject to ongoing cleaning, correction, and reclassification of onset dates as further data become available), reporting delays, and problems related to missing data (which may be non-random). In our current study, the hierarchical approach used by Canada's most populous province (Ontario) for replacement of missing data could have had distorting effects on measured disease epidemiology: the replacement of missing onset dates with dates of specimen collection could have resulted in the artifactual appearance of early turning points identified by our model, due to limitations in weekend staffing early in the outbreak. If, as we believe to be the case, public health laboratories did not have sufficient emergency staffing to keep up with testing on weekends such that weekend specimen log-ins declined sharply, this would have created the appearance of epidemic "fade out" on weekends. Other factors that might distort the apparent epidemiology of disease include changes in guidelines for laboratory testing of suspected cases, improved surveillance and public health alerts at later stages of the outbreak leading to increased case ascertainment or over-reporting of cases [20]. However, the quality of the time series will tend to improve with the duration of the epidemic, both because stochastic variation is "smoothed out", and also because small variations become less important as the cumulative series becomes longer. We note that a further application of the Richards model in the context of influenza would relate to comparison of the epidemiology of the 2009 influenza A H1N1 epidemic to past Canadian epidemics, though such an endeavor is beyond the scope of the present study.

In summary, we believe that the Richards model provides an important tool for rapid epidemic modeling in the face of a public health crisis. However, predictions based on the Richards model (and all other mathematical models) should be interpreted with caution early in an epidemic, when one need to balance urgency with sound modeling. At their worst, hasty predictions are not only unhelpful, but can mislead public health officials, adversely influence public sentiments and responses, undermine the perceived credibility of future (more accurate) models, and become a hindrance to intervention and control efforts in general.

## Competing interests

The authors declare that they have no competing interests.

## Authors' contributions

YHH conceived the study, carried out the analysis, and wrote the first draft. DF interpreted the results and revised the manuscript. JW participated in the analysis, the interpretation of results, and the writing. All authors read and approved the final manuscript.

## Supplementary Material

Additional file 1 **Electronic Supplementary Material**. 2009 Canada novel Influenza A(H1N1) daily laboratory-confirmed pandemic H1N1 case and hospitalization data. Click here for file
